# Supplementary material for: Impact of agile intuition on innovation behavior: Chinese evidence and a new proposal
Source: PLoS One. 2022 Apr 28;17(4):e0262426. doi: 10.1371/journal.pone.0262426 (PMC9049368; doi:10.1371/journal.pone.0262426)
Supplement: S1 Data — (ZIP) [file pone.0262426.s001.zip › Data/Questionnaire.docx]

[1] Your gender is

Men Women

1. Your age is

≤30 31-40 41-50 51-60 ≥61

1. Your degree is

Primary school and below Junior high school [Senior](C:/Users/ss19970417/AppData/Local/youdao/dict/Application/8.10.3.0/resultui/html/index.html#/javascript:;) [high](C:/Users/ss19970417/AppData/Local/youdao/dict/Application/8.10.3.0/resultui/html/index.html#/javascript:;) [school](C:/Users/ss19970417/AppData/Local/youdao/dict/Application/8.10.3.0/resultui/html/index.html#/javascript:;)

[Undergraduate](C:/Users/ss19970417/AppData/Local/youdao/dict/Application/8.10.3.0/resultui/html/index.html#/javascript:;) [course](C:/Users/ss19970417/AppData/Local/youdao/dict/Application/8.10.3.0/resultui/html/index.html#/javascript:;) Master degree or above

[4] Your position is

Front-line management personnel [Middle](C:/Users/ss19970417/AppData/Local/youdao/dict/Application/8.10.3.0/resultui/html/index.html#/javascript:;) [management](C:/Users/ss19970417/AppData/Local/youdao/dict/Application/8.10.3.0/resultui/html/index.html#/javascript:;)

[Senior](C:/Users/ss19970417/AppData/Local/youdao/dict/Application/8.10.3.0/resultui/html/index.html#/javascript:;) [management](C:/Users/ss19970417/AppData/Local/youdao/dict/Application/8.10.3.0/resultui/html/index.html#/javascript:;) [Grass-roots](C:/Users/ss19970417/AppData/Local/youdao/dict/Application/8.10.3.0/resultui/html/index.html#/javascript:;) [employees](C:/Users/ss19970417/AppData/Local/youdao/dict/Application/8.10.3.0/resultui/html/index.html#/javascript:;)

[5] Your company's industry is

[Manufacture](C:/Users/ss19970417/AppData/Local/youdao/dict/Application/8.10.3.0/resultui/html/index.html#/javascript:;) [Trade](C:/Users/ss19970417/AppData/Local/youdao/dict/Application/8.10.3.0/resultui/html/index.html#/javascript:;) [Financial](C:/Users/ss19970417/AppData/Local/youdao/dict/Application/8.10.3.0/resultui/html/index.html#/javascript:;) Traditional service [Architecture](C:/Users/ss19970417/AppData/Local/youdao/dict/Application/8.10.3.0/resultui/html/index.html#/javascript:;) other

1. The current location of your company is

East Central West Overseas

1. Your company was established at

1 year or less 1-5 years 5-10 years 10 years or more

1. The number of employees in your company is

No more than 1000 people From 1000 to 2000 people

From 2000 to 3000 people More than 3000 people

1. The nature of your company is

State-owned State-owned holding Private holding

Private sole proprietorship foreign company Other

1. Is your company is a high-tech enterprise

Yes No

1. Has your company ever received strategic guidance

Yes No

1. When I learn new knowledge, I will teach it to other colleagues

Completely disagree Compare disagree Disagree Uncertainty

Compare consent Consent In full agreement

1. If I get some information, I will also share it with my colleagues

Completely disagree Compare disagree Disagree Uncertainty

Compare consent Consent In full agreement

1. The techniques I have will also teach to other colleagues without reservation

Completely disagree Compare disagree Disagree Uncertainty

Compare consent Consent In full agreement

1. When I have questions asking my colleagues, they tell me what they know

Completely disagree Compare disagree Disagree Uncertainty

Compare consent Consent In full agreement

1. When I have something I don't understand at work and need to ask my colleagues, they will teach me

Completely disagree Compare disagree Disagree Uncertainty

Compare consent Consent In full agreement

1. Colleagues all agree with the culture of our company

Completely disagree Compare disagree Disagree Uncertainty

Compare consent Consent In full agreement

1. The company encourages everyone to share new information and establish a corresponding reward system

Completely disagree Compare disagree Disagree Uncertainty

Compare consent Consent In full agreement

1. Colleagues all love the company's culture and system

Completely disagree Compare disagree Disagree Uncertainty

Compare consent Consent In full agreement

1. Colleagues work in strict accordance with the rules and regulations

Completely disagree Compare disagree Disagree Uncertainty

Compare consent Consent In full agreement

1. The company has clear rules on everyone's work content

Completely disagree Compare disagree Disagree Uncertainty

Compare consent Consent In full agreement

1. How to have a set of system to spread the company

Completely disagree Compare disagree Disagree Uncertainty

Compare consent Consent In full agreement

1. The company's products need the joint efforts of everyone to complete

Completely disagree Compare disagree Disagree Uncertainty

Compare consent Consent In full agreement

1. The degree of coordination between the company's various departments is very high

Completely disagree Compare disagree Disagree Uncertainty

Compare consent Consent In full agreement

1. The company provides training or rotation methods to improve the cooperation ability of personnel

Completely disagree Compare disagree Disagree Uncertainty

Compare consent Consent In full agreement

1. We will get support from other departments when our work needs it

Completely disagree Compare disagree Disagree Uncertainty

Compare consent Consent In full agreement

1. The company has recently improved the quality of its products or services through technology or management innovation

Completely disagree Compare disagree Disagree Uncertainty

Compare consent Consent In full agreement

1. The company has accelerated the launch of new products or services through innovation

Completely disagree Compare disagree Disagree Uncertainty

Compare consent Consent In full agreement

1. The company improves the profitability of its products or services through innovation

Completely disagree Compare disagree Disagree Uncertainty

Compare consent Consent In full agreement

1. Innovation can reduce the research and development costs of products or services

Completely disagree Compare disagree Disagree Uncertainty

Compare consent Consent In full agreement

1. The company has introduced a new process equipment or management system, and has accelerated the update speed of products or services

Completely disagree Compare disagree Disagree Uncertainty

Compare consent Consent In full agreement

1. Enterprises have invented new technologies or new management system through innovation, and optimized the operation process

Completely disagree Compare disagree Disagree Uncertainty

Compare consent Consent In full agreement

1. We will respond to changing market demand

Completely disagree Compare disagree Disagree Uncertainty

Compare consent Consent In full agreement

1. We respond faster to changes in market demand than our major competitors

Completely disagree Compare disagree Disagree Uncertainty

Compare consent Consent In full agreement

1. We will find some strategies for the company that do not work

Completely disagree Compare disagree Disagree Uncertainty

Compare consent Consent In full agreement

1. We are able to summarize the causes of the strategic failure

Completely disagree Compare disagree Disagree Uncertainty

Compare consent Consent In full agreement

1. We are able to learn from the wrong strategies and make less mistakes

Completely disagree Compare disagree Disagree Uncertainty

Compare consent Consent In full agreement

1. When we know which strategies are effective and which strategies are ineffective, we adjust our strategies regularly and take action

Completely disagree Compare disagree Disagree Uncertainty

Compare consent Consent In full agreement

1. Our company attaches great importance to my opinion, and will make strategic adjustments according to my opinion

Completely disagree Compare disagree Disagree Uncertainty

Compare consent Consent In full agreement

1. When we find that it does not work, we adjust our strategic goals and find alternative strategies

Completely disagree Compare disagree Disagree Uncertainty

Compare consent Consent In full agreement
