# Supplementary material for: Impact of agile intuition on innovation behavior: Chinese evidence and a new proposal
Source: PLoS One. 2022 Apr 28;17(4):e0262426. doi: 10.1371/journal.pone.0262426 (PMC9049368; doi:10.1371/journal.pone.0262426)
Supplement: Attachment — Submitted filename: review.doc [file pone.0262426.s002.doc]

I am honored to be invited to review this manuscript of Chinese studies. This research has greater practicality, and I can see the authors have done lots of work to get to its data and conclusions. I have offered some comments from reading the paper below. I wouldn't consider myself as an expert in Agile Intuition, I apologize for any comments that I might have raised as a consequence of my lack of knowledge in this area.

1. The introduction part introduces the important influence of the Agile Intuition of top managers on the innovation behavior of enterprises. The research questions are derived from the problems and the status quo, with a rigorous logic and hierarchy. But Line 99, it is not very appropriate to directly give the influence of KS and SL on innovation behavior, without any foreshadowing and presentation of related concepts and researches. Here readers do not know what KS and SL are.

2. In the part of literature review and hypothesis, we can understand the impact of AI on IB, as well as the possible mediating role of KS and moderating role of SL. However, for Hypothesis 5: SL moderates the positive effect of AI on IB, is there more support and literature to explain it?

3. In the section of sample and data collection, authors distribute and collect two sets of questionnaires to middle- and high-level managers and heads of subsidiaries to measure AI and SL, respectively. Than three sets of questionnaires were distributed and collected to measure SL, KS, and IB. So, how is the SL questionnaire measured, and how many times does it measure? Does the group of subjects change? Please state it more clearly.

4. Following question 3, the research object is the AI of top managers, involves SL of top managers and KS with employees. We see Line 235: there are 112 middle and senior managers and 356 subsidiary managers. Although there are only 305 valid samples, but in Line 240 and Table 1: 74.43% are ordinary staff, and 25.57% were middle and senior managers. Where do ordinary employees come from? Please explain and standardize the subject group in detail.

5. Regarding the phenomenon in Figure 2, why does this result occur, is it related to the Chinese cultural background or is it universal? Please give a detailed explanation and description.

6. This study conducts a relatively sufficient data analysis on the hypothesis, but there are still shortcomings in the discussion. For examples, data analysis supports Hypothesis 5, but how about its theoretical analysis and discussion? Authors need more discussion to reach the 3 conclusions in line 416-418. Please supplement more analyze and discuss the impact of the results of previous section from various aspects to support the conclusions of this article, not just restate the research results.
